# Dyadic differences in empathy scores are associated with kinematic similarity during conversational question–answer pairs

**DOI:** 10.1080/0163853X.2025.2467605

**Published:** 2025-02-26

**Authors:** James P. Trujillo, Rebecca M. K. Dyer, Judith Holler

**Affiliations:** a Donders Institute for Brain, Cognition & Behaviour, Radboud University, Nijmegen, The Netherlands; b Max Planck Institute for Psycholinguistics, Nijmegen, The Netherlands; c Institute for Logic, Language & Computation, University of Amsterdam, Amsterdam, The Netherlands; d Centre for Human Developmental Science, Cardiff University, Cardiff, UK

## Abstract

During conversation, speakers coordinate and synergize their behaviors at multiple levels, and in different ways. The extent to which individuals converge or diverge in their behaviors during interaction may relate to interpersonal differences relevant to social interaction, such as empathy as measured by the empathy quotient (EQ). An association between interpersonal difference in empathy and interpersonal entrainment could help to throw light on how interlocutor characteristics influence interpersonal entrainment. We investigated this possibility in a corpus of unconstrained conversation between dyads. We used dynamic time warping to quantify entrainment between interlocutors of head motion, hand motion, and maximum speech *f0* during question–response sequences. We additionally calculated interlocutor differences in EQ scores. We found that, for both head and hand motion, greater difference in EQ was associated with higher entrainment. Thus, we consider that people who are dissimilar in EQ may need to “ground” their interaction with low-level movement entrainment. There was no significant relationship between *f0* entrainment and EQ score differences.

When engaging in conversation with one another, behaviors, such as the way we move or speak, often converge between interlocutors. This process, called interpersonal entrainment, is thought to facilitate successful communication, due to its association with various positive social outcomes (Bowsher-Murray et al., 2023; Hove & Risen, 2009; Rabinowitch & Meltzoff, 2017; Tarr et al., 2015; Vacharkulksemsuk & Fredrickson, 2012; Zampella et al., 2020). However, there is much variation in the extent to which we entrain our behaviors with our conversational partner. This may depend on the specific communicative context, or individual differences. One possibility is that individuals who are more similar in their social dispositions may find it easier to entrain their behaviors with one another. While there is recent interest in the idea of social dispositions as an influencing factor on social interaction (i.e., the double-empathy problem (Milton, 2012; Milton et al., 2022)), it is currently not known whether or how differences in traits that are relevant for social interaction, such as empathy, impact the extent of behavioral entrainment during conversation.

Recent studies of conversational behavior demonstrate that speakers form dynamical patterns of interaction and behavior coordination with one another. In particular, many studies have investigated entrainment, quantifying the extent to which two interacting individuals exhibit similar behaviors, either at the same time or with some lag. In speech, entrainment can refer to linguistic entrainment, such as the use of similar words, syntactic structure, or even converging on a particular topic (Duran et al., 2019), or acoustic entrainment, such as converging on similar prosodic features (Buder & Eriksson, 1997). Similarly, there may be kinematic entrainment, such as moving the hands or head similarly to one’s conversational partner (Danner et al., 2021; Louwerse et al., 2012; Trujillo et al., 2023), or gestural entrainment in the form of using similar types of gestures to refer to the same concept (Cienki et al., 2014; Holler & Wilkin, 2011; Holler et al., 2022; Kimbara, 2006; Rasenberg, Dingemanse, et al., 2020). While some earlier studies of kinematic entrainment focused on moment-to-moment behavioral similarity, or so-called synchrony of behavior, we also observe linguistic and kinematic entrainment when comparing sequences of conversational turns, indicating that speakers entrain their movements to one another in fluctuation with the back and forth of dialog. Importantly, entrainment does not necessarily occur across all levels or modalities but. rather, there seems to be a dynamic interplay between different forms of entrainment (Ostrand & Chodroff, 2021; Rasenberg, Özyürek, et al., 2020; Rasenberg et al., 2022; Trujillo et al., 2023). These dynamically emerging patterns of entrainment are thought to facilitate interaction through allowing speakers to converge on shared meaning, resolve ambiguities, elaborate and progress the conversation (Healey et al., 2014; Oben & Brône, 2016) and the emergence of such patterns is generally associated with positive affiliation with one’s partner (Hu et al., 2022; Rennung & Göritz, 2016). The term “entrainment” has been measured and defined in different ways, such as on very different time-scales (e.g., some studies use entrainment to mean similarity in behavior across an entire interaction; whereas, others refer to similarity of behaviors between adjacent turns; Wynn & Borrie, 2022). Importantly, entrainment occurs along a continuum, whereby entrainment between two individuals may be high, indicating that their behavior is highly similar to one another, or it may be low, indicating that their behavior is highly dissimilar. Low entrainment has also been referred to as “disentrainment,” and at least for speech prosody is also associated with positive evaluation of conversation (Pérez et al., 2016).

## Interpersonal differences and entrainment

While entrainment is generally seen as a positive aspect of social interaction, individuals may differ substantially in the extent to which they achieve effective coordination (both in terms of entrainment and complementarity). Thus, interpersonal differences appear to play a role. This has primarily been demonstrated by differences between autistic and nonautistic people in entrainment. For example, autistic people, while demonstrating linguistic entrainment during interactions with autistic or nonautistic people in structured tasks (Branigan et al., 2016; Slocombe et al., 2013), demonstrate differences compared with nonautistic people in linguistic entrainment during spontaneous conversation (Bottema-Beutel, 2017). A similar pattern emerges across various modalities of interpersonal entrainment, with conversations involving autistic people demonstrating less entrainment of bodily motion (Georgescu et al., 2020; Noel et al., 2018; Romero et al., 2018) and prosody (Lehnert-LeHouillier et al., 2020; Ochi et al., 2019) compared with conversations involving only nonautistic people. This pattern has also been observed outside of conversation in a tacit communication game, with autistic adults being less likely than nonautistic adults to entrain their nonverbal communicative strategies to that of their partner. Notably, this occurred despite a similar desire and ability to communicate in the task (Wadge et al., 2019).

Importantly, these differences in interpersonal entrainment may not be specific to the autistic population per se. Zampella et al. (2020), as in the studies already described, found that, compared with nonautistic youth, autistic youth demonstrated lower coordination of reciprocal smiling in dyadic conversations with their mothers and with an unfamiliar adult. However, the level of smile coordination, for both autistic and nonautistic participants, was associated with measures of social ability (Social Responsiveness Scale-2 (SRS-2); Constantino & Gruber, 2012) and dispositional empathy (Interpersonal Reactivity Index (IRI); Davis, 1983), with higher smile coordination occurring in children with higher (parent-reported) empathy. The observed reduction in interpersonal entrainment in autistic people, therefore, may be transdiagnostic—related to characteristics present in but not specific to this population. This is further supported by McNaughton and Redcay (2020), whose review of interpersonal synchrony in autistic participants found that intrapersonal characteristics, such as motor timing, may mechanistically explain the observed entrainment differences in this population. Thus, taking a transdiagnostic approach and investigating the relationship between the characteristics highlighted here, such as empathy (Zampella et al., 2020) and interpersonal entrainment, is necessary to improve our understanding of the mechanisms of interpersonal differences in entrainment.

This is of further relevance, as such a relationship between interpersonal entrainment and empathy has also been described by Fujiwara and Daibo (2022), who found that empathic accuracy (in this case, the ability to classify the thoughts or feelings of an interaction partner from a video recording) is positively correlated with interpersonal entrainment in the form of postural mirroring. Importantly, Zampella et al. (2020) and Fujiwara and Daibo (2022) looked at the empathy of only one individual. Rather than coordination being linked to the magnitude of empathy scores in one individual, it may be that interpersonal coordination is more generally related to the difference in empathy scores between two individuals. This would be more in line with the fact that interpersonal synchrony does not rely on the efforts of one individual or the other but rather the dynamic coupling of two (or more) individuals’ behaviors (Schmidt et al., 2012).

### Variation in interpersonal entrainment as a two-person problem

Understanding variation in interpersonal entrainment will require us to consider that entrainment arises from the interaction between individuals. Assessing variation in the extent to which two individuals entrain with one another or show behavioral similarity should, therefore, consider the impact of interpersonal differences between entraining interlocutors.

The relationship between interpersonal differences and social behaviors has been described by the double-empathy problem: the idea that people, as a rule, find it more difficult to empathize with those who differ from them than with those who are similar to them (Milton, 2012; Milton et al., 2022). This has been most famously applied to communication breakdown between autistic and nonautistic individuals due to differences in diagnostic status. Such a theoretical reduction in empathy between autistic and nonautistic individuals may negatively impact aspects of social interaction, with Williams et al. (2021) finding that flow, rapport, enthusiasm, and mutual affect were greater in conversations between two autistic adults than in conversations between autistic and nonautistic adults. While the double-empathy problem does not directly refer to empathy traits specifically, the overall framework seems to fit well with previous research suggesting a link between empathy and motor synchrony (Fujiwara & Daibo, 2022; Zampella et al., 2020). At the same time, thinking of interpersonal coordination as a two-person “problem” (Bolis et al., 2018; Schilbach, 2016) and within the framing of interpersonal differences as relating to our ability to connect with one another (Milton, 2012; Milton et al., 2022) suggests that we should consider differences in trait scores, such as empathy, rather than the magnitude of one individual’s score.

The impact of such interlocutor differences on interpersonal entrainment in conversations has been investigated. Georgescu et al. (2020) investigated interpersonal entrainment in conversations involving two autistic individuals, involving one autistic and one nonautistic individual, or involving two nonautistic individuals. While these researchers found that conversations containing at least one autistic interlocutor demonstrated less interpersonal entrainment than dyads containing only nonautistic participants, interpersonal entrainment did not significantly differ between dyads containing participants with matching diagnostic status and those containing participants with mismatched diagnostic status. While an interesting study to systematically compare autistic, nonautistic, and mixed dyads, Georgescu et al. (2020) did not account for interpersonal differences beyond broad neurotype classification. This is particularly important following neurodiversity theories that humans diverge in multiple ways that cannot be captured by diagnostic status alone (Singer, 2016). Furthermore, the double-empathy problem is not only relevant to autistic people but to broader interpersonal differences (Milton et al., 2022). Therefore, it appears a logical next step to investigate whether interpersonal differences in empathy—a transdiagnostic trait observed to relate to interpersonal entrainment (Fujiwara & Daibo, 2022; Zampella et al., 2020)—relates to levels of interpersonal entrainment. Such a relationship could contribute to understanding the mechanisms of interpersonal differences in entrainment. This transdiagnostic approach, focusing more on individual differences, also aligns well with recent work that frames social interaction difficulties (such as in autism) as involving a misattunement between individuals rather than any deficiency in a particular group (Bolis et al., 2018). From this perspective, the most informative way forward is to assess whether we can determine whether there are measures of interpersonal differences that, while collected individually, are predictive of behavioral measures of attunement, or coupling, during conversation.

### The present study

The current study aims to better elucidate how variation in interpersonal behavioral entrainment may arise from differences in conversational partners. The idea that partner differences may be associated with interpersonal behavioral (dis)similarity largely builds on the double-empathy problem as a general framework for understanding differences in social interaction dynamics, as well as the findings of a significant relationship between interpersonal entrainment and the strength of autistic traits (Chen et al., 2022; Cheng et al., 2017). More specifically, the present study aimed to determine whether interpersonal behavioral entrainment is associated with differences in empathy quotient (EQ) score between partners engaged in naturalistic, unscripted dyadic conversation.

In the present study, we analyzed 34 video and audio recordings from the Communication in Action (CoAct) corpus (previously analyzed in Nota et al., 2021; Ter Bekke et al., 2024; Trujillo & Holler, 2021) in which nonautistic Dutch speakers engaged in three dyadic 20-minute conversations. We additionally recorded empathy scores based on the EQ questionnaire, and calculated empathy-score differences. From the conversation data, question–response pairs (which form a core coordinative structure and building block of everyday conversational interaction) from each dyad were assessed for interpersonal behavioral entrainment. To capture the multimodal nature of interpersonal behavior, three measures of behavioral entrainment were investigated: head entrainment, hand entrainment, and prosodic matching. We specifically measured what Wynn and Borrie (2022) refer to as *static local proximity* entrainment, calculating the similarity in movement or prosody values between adjacent speaking terms. Movement similarity was used to quantify entrainment for both motor measurements (head and hand), as we made use of dynamic time warping (DTW) to compare kinematic profiles between interlocutors. Utilizing DTW allowed us to compare kinematic profiles between questions and responses, which may differ in length, rather than focusing on small, fixed window sizes inherent in cross-correlation methods. We hypothesized that lower behavioral entrainment would be associated with higher partner EQ differences, which would suggest that behavioral entrainment is lower when conversational partners are able to empathize with one another to a similar extent. Understanding whether differences in empathy affect interaction dynamics during natural conversation will further inform whether interpersonal differences play a role in entrainment and, in particular, whether the patterns expected by the double-empathy problem are relevant for such investigations.

## Methods

### Data availability and open science

We report all measures and exclusions utilized in the current study. Materials used in this study, including data-analysis scripts and preprocessing scripts, are available via the Open Science Framework (OSF) at the following link: https://osf.io/hp48e/.

Note that the raw data, including audio and video files, cannot be made public due to ethical constraints and related concerns for privacy and anonymity.

The current study was not preregistered, and a priori sample-size estimation could not be performed, as this was a follow-up study using a corpus that had already been collected as part of a larger project (see subsection *Materials*).

### Materials

Data for the present study consisted of video, audio, and questionnaire data collected as part of the Communication in Action (CoAct) Corpus (Nota et al., 2021; Ter Bekke et al., 2024; Trujillo & Holler, 2021). The CoACT Corpus consists of 59 dyads (37 female–female, 10 male–male, 12 male–female) of acquainted native Dutch-speakers conversing. For 34 of these dyads (68 participants, 51 female, mean age 23.10 years) question–response and social action coding was completed, and thus these dyads were included in the present study. Each dyad engaged in three 30-minute Dutch conversations: the first a natural conversation, the second a discussion of opinions about predefined topics, and the third a collaborative planning task. Recordings were made in a soundproof room at the Max Planck Institute for Psycholinguistics in the Netherlands. Two cameras filmed the front of each participant, while two more filmed each of their bodies from a 45-degree angle and two others recorded a birds-eye view. Finally, one camera filmed the two participants together. All cameras filmed at 25 frames per second. Each participant’s speech was recorded by a directional microphone. All video and audio files were synchronized, leaving a time resolution of 40 ms. Both video and audio files were utilized for the present study. Following the conversation tasks, participants completed two questionnaires: the empathy quotient (EQ; Baron-Cohen & Wheelwright, 2004; Groen et al., 2015) and the brief version of the Fear of Negative Evaluation Scale (Leary, 1983; Watson & Friend, 1969). Responses to the EQ were used for the present study.

The EQ is a 60-question self-report questionnaire, consisting of 40 questions designed to gauge the respondent’s empathy skills and 20 filler questions. It is scored out of 80, as participants can achieve up to two points on each of the target questions (Baron-Cohen & Wheelwright, 2004). The Dutch version of this questionnaire has also shown good internal consistency (Cronbach’s α = 0.89; Groen et al., 2015). It is important to note here that there is some debate about how to classify empathy (Kerem et al., 2001), with researchers identifying different types of empathy. However, it is widely reported that there are at least two types—cognitive and affective (Davis, 1980)—with cognitive empathy referring to the recognition of others’ mental states and affective empathy referring to a sympathetic emotional response (Baron-Cohen & Wheelwright, 2004). The EQ does not distinguish between types of empathy.

The present study was conducted within existing ethical approval for a larger project, approved by the ethics committee of the social sciences department of Radboud University, Nijmegen (approval code: ECSW 2018-124).

### Question–response pair annotations

Rather than analyzing all turn segments within the corpus, we focused on question–response pairs, as questions are incredibly common in conversation and since questions and their responses are so-called *adjacency pairs* (Schegloff, 2007). Adjacency pairs provide a useful focus for these analyses, as such focus allows us to zoom in on strongly reciprocal moments of the interactions requiring a high degree of coordination. In other words, the overall behavior of Speaker A will be related to the behavior of Speaker B. This clear question–response format therefore avoids some of the noise typically created by the varying contexts of turn-taking in conversation. As described in Nota et al. (2021) and Trujillo and Holler (2021), questions and responses were manually annotated using the following procedure. First, we made an automatic orthographic transcription of the speech signal using the Bavarian Archive for Speech Signals Webservices (Kisler et al., 2017). Questions were identified and coded in ELAN (5.5; Wittenburg et al., 2006), largely following the coding scheme of Stivers and Enfield (Stivers & Enfield, 2010). In addition to this scheme, more rules were applied on an inductive basis to account for the complexity of the data in the corpus. Specifically, we adopted a holistic approach that took into consideration visual bodily signals, context, phrasing, intonation, and addressee behavior. Nonverbal sounds were excluded (e.g., laughter, sighs). This was done by two human coders, one native speaker of Dutch, and one highly proficient speaker of Dutch. Interrater reliability between the two coders was calculated with raw agreement (Cohen, 1960; Landis & Koch, 1977) on 12% of the total data (four dyads, all tasks). A standard overlap criterion of 60% was used. Reliability between the coders resulted in a raw agreement of 75% for questions and a raw agreement of 73% for responses, indicating substantial agreement. This resulted in a total of 6,778 questions (duration Mdn = 1,114 ms, range = 99–13,145 ms, IQR = 1,138 ms). As not all questions received a response, the present analyses are based on a subset of 4,436 question–response sequences. Note that other levels and types of annotations were made for this corpus (see, e.g., Nota et al., 2021; Trujillo & Holler, 2021), as it is part of a much larger project. However, these other annotations were not used for the present study.

### EQ score differences

**Figure 1.**
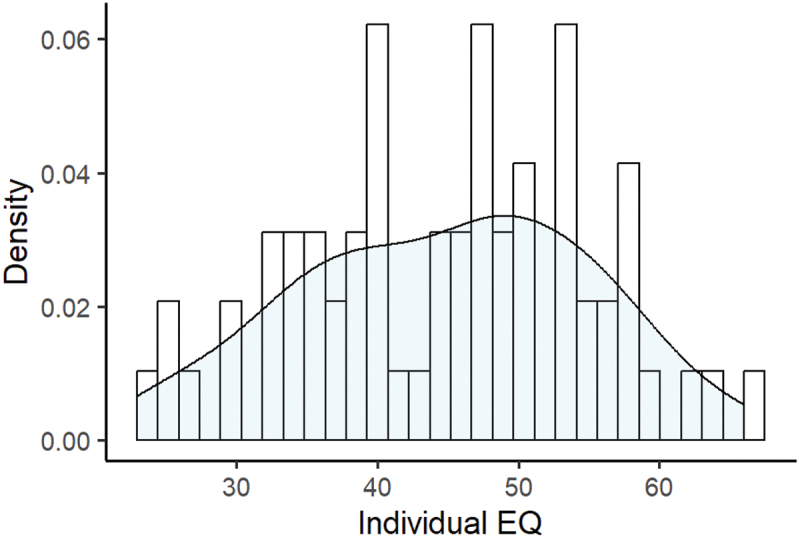
Distribution of individual EQ scores. EQ is given along the *x*-axis, while the density of distributions is given on the *y*-axis. The plot provides both a histogram and a density curve of these values.

While all of the participants completed the EQ, in only 19 dyads did both partners complete every question. Following the procedure outlined by Lawrence et al. (2004) for handling missing values within the EQ, scores for questions that were skipped by participants were replaced with the mean score of the group for that question, rounded to the closest integer (Lawrence et al., 2004). See Figure 1 for the distribution of individual EQ values. Following this handling of the missing values, the absolute difference between EQ scores of partners in each of the 34 dyads was calculated and taken as the final measure of EQ score differences.

### Kinematic entrainment

To calculate motor similarity, we used OpenPose (Qiao et al., 2017) motion tracking to extract the two-dimensional motion of the nose, right-hand, and left-hand key points from each participant. Motion data were extracted for each question and response annotation (described above). We used motion of the nose as a measure of head movement.

**Figure 2.**
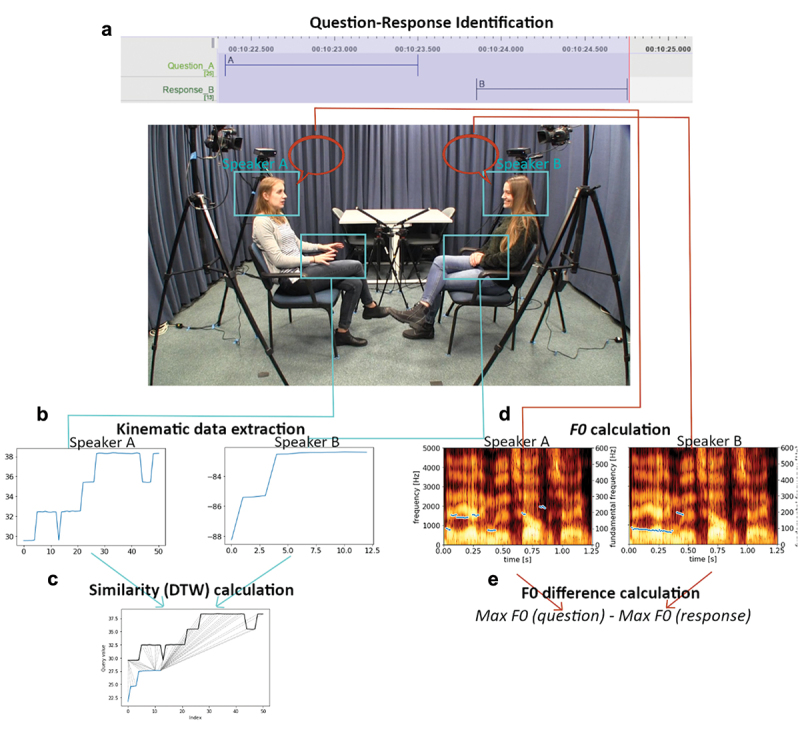
Overview of data and processing steps. In the center, a still frame is presented of the data recording set-up. The procedure involves qualitative, manual annotation (depicted above the still frame), and quantitative extraction of kinematic and acoustic features (depicted below the still frame). As a first step, question–response sequences were identified through manual coding of the data (Panel A). Motion data were extracted from the head and hands, providing displacement time series for each articulator during each question or response segment (Panel B). These motion data were compared using dynamic time warping (DTW) to provide a similarity score (Panel C). Audio recordings were used to extract the pitch contour (Panel D) for each question and response segment. Our prosodic matching score was calculated as the absolute difference in maximum F0 (pitch) between the question and response (Panel E). Participants depicted in this figure gave written, informed consent for their image to be used in academic publications.

Entrainment was then calculated based on the similarity between movement produced during the question (by the questioner) and movement during the response (by the responder). Specifically, head and hand asynchrony were calculated using dynamic time warping (DTW). DTW works by using a warping algorithm to nonlinearly align time series while preserving the order of time points in the two time series. After finding a maximal spatial alignment between the two time series, the remaining error, or distance, between the two is taken as a measure of dissimilarity (see Figure 2c). This distance score is normalized for the time-series lengths, ensuring that the distance score is not influenced by differences in time-series length. It is important to note that it is not possible to say whether there is or is not entrainment between two time series from just the distance score. Instead, these scores provide a magnitude of entrainment, where warping two completely unrelated time series will likely result in a very high value (i.e., high dissimilarity) and warping two highly similar time series (e.g., two highly entrained time series) will result in a much lower value. Rather than interpreting the values themselves, it is therefore important to use the values as contrasts between conditions or to test for a linear relationship between distance (henceforth, entrainment) values and some other variable of interest.

The DTW approach differs from other measures of interpersonal entrainment such as cross-recurrence quantification analysis (CRQA; e.g., Fusaroli & Tylén, 2016; Louwerse et al., 2012) in a crucial way. Methods such as CRQA are intended to capture the overall dynamics of recurring patterns of behavior between individuals. For example, the stability and complexity with which two individuals match their behaviors (e.g., both producing a gesture within a short time of one another). Critically, these analyses are focusing on the occurrence of matched behavior events. In contrast, DTW is not quantifying recurring patterns over the course of an interaction—or even capturing the presence of behavior events such as gestures. Instead, we use it to quantify the similarity between two manually annotated events (see also Pouw & Dixon, 2020; Pouw et al., 2021; Trujillo et al., 2023 for similar implementations).

In the case of kinematic asynchrony, DTW therefore calculates the distance between the motion time series during the asking of a question and the motion time series during the uttering of the corresponding response. This distance was taken as a measure of similarity between each other’s movements, wherein low values reflect high similarity (i.e., minimal warping to match the time series). DTW was conducted for nose movements, left-hand movements, and right-hand movements between partners for all question–response pairs in each dyad. This was performed in Python using Giorgino’s (2009) DTW Python package. The mean similarity of the right and left hand was taken as the final measure of hand-movement similarity. Nose movement was used to calculate head-movement similarity. These similarity scores are then taken as a measure of kinematic entrainment. See Figure 2 for an overview of processing steps.

### Prosodic mismatch

To calculate prosodic dissimilarity, mean fundamental frequency (pitch), between 75 and 300 Hz, was first calculated using the Python package Parselmouth PRAAT (Boersma & Weenink, 2007; Jadoul et al., 2018) for each question and response. We then calculated the difference in F0 between the question and corresponding response. This method was inspired by Levitan and Hirschberg (2011) and Edlund et al. (2009), who calculated a correlation coefficient for each dyad based on F0 of adjacent speech turns. We use absolute difference in order to utilize the full distribution of question–response pair data in a mixed-model approach (see below). See Figure 2 for an overview of processing steps.

### Analyses: assessing interpersonal entrainment in question–response pairs

Before assessing whether interpersonal behavioral dissimilarity is related to other factors, we first assessed whether there was any evidence for different-than-chance-level dissimilarity. This was done by comparing the observed question–response dissimilarity against shuffled data, wherein the question–response correspondence was broken. To this end, we shuffled the question–response correspondences within each dyad, so that each question was paired with a response different from that which it had originally corresponded with. By keeping the shuffling within dyads, we also keep any intrinsic level of movement (dis)similarity that may exist between two interlocutors. Our test is thus aimed at determining whether question–response sequences show less dissimilarity (i.e., greater degree of entrainment) than we would expect from any intrinsic movement (dis)similarity occurring simply by being engaged in dialogue. This surrogate test procedure thus applies the same logic as the standard test for greater-than-chance synchrony (Delaherche et al., 2012), but here applied to dissimilarity. We then built separate models for each of the three dissimilarity variables, with dissimilarity (i.e., head asynchrony, hand asynchrony, prosodic dissimilarity) being the dependent variable and a random intercept for ID, which identified the specific dyad and participant (included a nested term for dyad and participant that led to convergence and singularity issues with model fit. A null model containing only the dependent variable and the random term was then compared, using a likelihood ratio test, against a full model that also contained data set (real versus shuffled) as a fixed effect. A significant model comparison indicates that dissimilarity is different from what would be expected by chance (i.e., in the shuffled data). Holm’s correction for multiple comparisons was applied to avoid the increased risk of a Type 1 error (Chen et al., 2017) associated with these three tests. Specifically, following the guidelines of Rubin (2021), Holm’s correction was applied to the two kinematic tests (i.e., head asynchrony and hand asynchrony).

### Analyses: assessing associations between EQ differences and interpersonal entrainment

To determine whether our measures of interpersonal behavioral dissimilarity were associated with differences in EQ scores, we utilized mixed effects linear regression models, implemented using the lme4 package in R (Bates et al., 2015). We built separate models for each of the three dissimilarity variables, with dissimilarity (i.e., head asynchrony, hand asynchrony, prosodic dissimilarity) as the dependent variable and a random intercept for ID, which identified the specific dyad and participant. A null model containing only the dependent variable and the random term was then compared, using a likelihood ratio test, against a full model that also contained EQ difference as a fixed effect. A significant model comparison indicates that adding the EQ difference explains significantly more variance in the dissimilarity data than is explained by interindividual variability (i.e., the random intercept) alone.

Alpha thresholds were adjusted to account for the increased false positive rate associated with performing three comparisons (Chen et al., 2017). Based on the guidelines of Rubin (2021), we categorize our tests as falling into families: EQ association with prosody and EQ association with kinematics. This follows from the guidelines of Rubin (2021) and is based on our hypotheses that relate to prosody and kinematics separately. We then calculate adjusted *p*-values for our head and hand kinematic models based on Holm’s method for multiple comparisons correction (Chen et al., 2017; Holm, 1979).

### Post hoc analysis: assessing associations between entrainment values

Besides behavior entrainment being related to (differences in) individual traits, entrainment in one modality may also be negatively correlated with entrainment in another modality (Trujillo et al., 2023). This would impact the way we must interpret why there may be associations between entrainment and EQ-differences in one modality or articulator and another. We therefore calculated partial correlations between each pair of entrainment variables while excluding the effect of the third entrainment variable. This was done using the partial.r function of R package psych (Revelle, 2020).

## Results

### Results: significance of interpersonal behavioral entrainment

For our comparison with the pseudo– (i.e., randomly matched) question–response pairs, we find that head asynchrony (χ^2^(1) = 61.129, *p* < .001), hand asynchrony (χ^2^(1) = 8.807, *p* = .003), and prosodic matching (χ^2^(1) = 8.190, *p* < .001) all significantly differed in the real question–response pairs compared to the pseudo-pairs. This indicates that the dissimilarity that we observe is different from what would be expected in chance circumstances. Specifically, we see higher kinematic asynchrony (i.e., lower similarity) in the real question–response pairs compared to the pseudo-pairs (head asynchrony *t*-value = 7.84; hand asynchrony *t*-value = 2.969) but lower F0 difference (i.e., higher prosodic matching) in the real pairs compared to the pseudo-pairs (*t*-value = 2.862).

These results indicate that all behavioral similarity measures show a difference from the values that would be expected in chance circumstances and, specifically, that kinematic entrainment is lower than would be expected in chance circumstances, while prosodic matching is higher than would be expected in chance circumstances.

### Results: associations between EQ differences and behavioral entrainment

**Figure 3.**
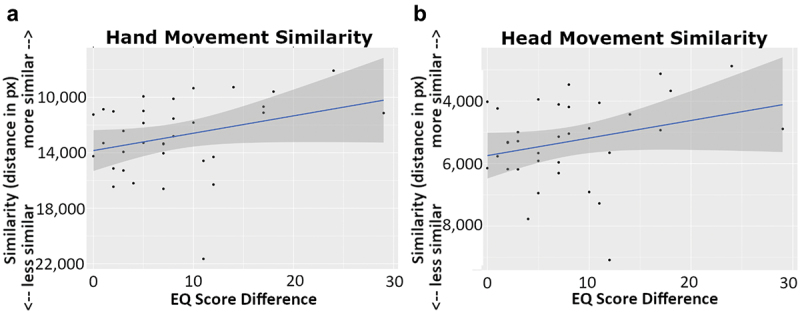
Scatterplots of the per-dyad average movement similarity values plotted against EQ score differences. The left panel (a) shows hand-movement similarity and the right panel (b) shows head-movement similarity. In both panels, similarity (measured as Euclidean distance in pixels) is given on the *y*-axis and EQ score difference (absolute difference) is given on the *x*-axis. A linear fit line is given in blue, with the gray shaded area indicating the 95% confidence band around the fit line.

For kinematic entrainment, we found that hand movement similarity was significantly positively associated with EQ difference (χ^2^(1) = 4.295, *p* = .038, Holm-corrected *p* = .044) such that for each additional point difference in EQ, hand similarity increased by 116.880 ± 50.67 (*t* = 2.307). See Figure 3a for an overview of these results.

Similarly, we found that head movement similarity was similarity positively associated with EQ difference (χ^2^(1) = 5.264, *p =* .022, Holm-corrected *p* = .044) such that for each additional point difference in EQ head movement similarity increased by 52.436 ± 25.28 (*t* = −2.074). See Figure 3b for an overview of these results.

We found no evidence for prosodic mismatch being associated with EQ difference (χ^2^(1) = 0.158, *p* = .691).

These results indicate that higher kinematic entrainment was associated with higher EQ score differences.

### Post hoc results: assessing associations between entrainment values

**Figure 4.**
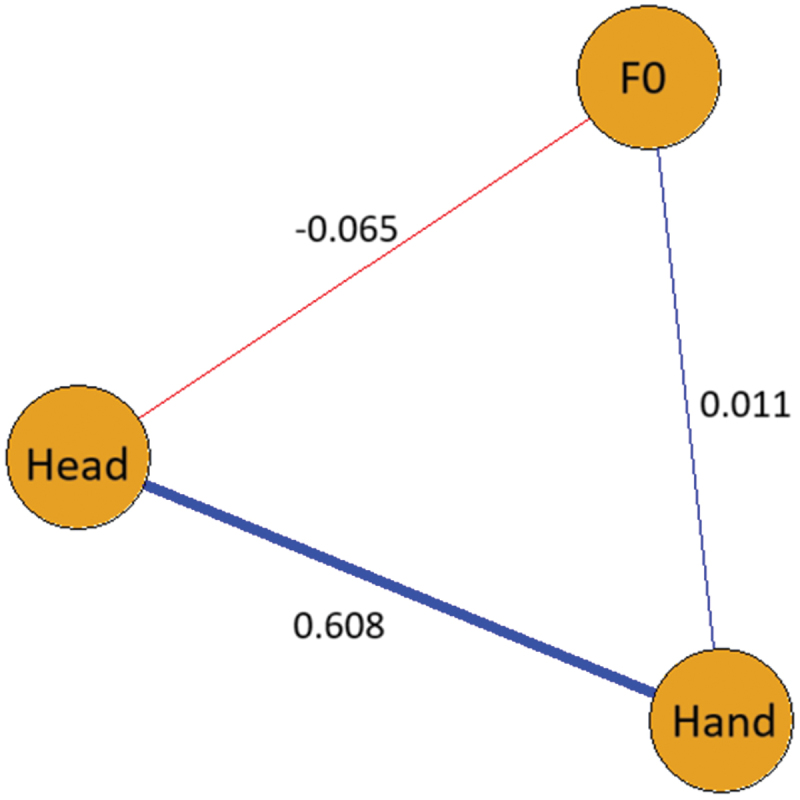
Associations between entrainment variables. Each variable is represented by a node in the graph, with the line (i.e., vertex) between two nodes representing the association (i.e., partial correlation) between the two variables. Stronger absolute associations are indicated by thicker vertices; whereas, direction of association is indicated by color (i.e., blue for positive, red for negative). Correlation coefficients are indicated next to each vertex.

Our exploratory post hoc analysis showed that only entrainment of the head and hands shows a strong association. Much weaker associations are found between F0 difference and either head or hand kinematic entrainment. See Figure 4 for a visualization of these associations.

## Discussion

Following the double-empathy problem, the present study aimed to investigate whether interpersonal behavioral entrainment during question–response sequences is associated with difference in EQ scores during dyadic conversation. We found that dyads with higher differences in EQ show higher levels of kinematic entrainment of the head and hands.

We first used a pseudo-pair analysis to determine whether behavioral entrainment was significantly different from chance. This was done because different-than-chance entrainment shows that we are measuring something that captures the interactive dynamic of conversation. This first analysis showed that the kinematic and prosodic entrainment observed in our data is significantly different from what would be observed in chance circumstances. However, kinematic entrainment was found to be lower than what would be expected by chance. Although this at first seems counterintuitive, absent or lower-than-chance entrainment has previously been observed, specifically, in relatively unconstrained dialogue (Healey et al., 2014; Howes et al., 2010). Additionally, our analyses focused on question-response pairs, which have an inherent asymmetry between the speakers—that is, speakers are actively responding to an imbalance of knowledge, an ambiguity, or are expressing a particular stance that is likely to elicit a response (e.g., expressing criticism by asking “Do you really think that’s a good idea?”). These results are therefore informative on several levels. First, we can be confident that we are observing true coordination between speaker behavior and for one of the core structures prevalent in everyday conversation. Second, our results support the notion that divergence, which may be a form of complementarity, is an important aspect of communicative coordination (Fusaroli et al., 2014; Healey et al., 2014).

The finding of lower-than-chance prosodic mismatch (i.e., high prosodic *matching*) is also interesting in light of work from Syrdal and Kim (2008), who measured F0 range across various interrogative (such as wh- questions), imperative, assertive, and affective speech acts. They found that F0 differed significantly across the various speech acts (Syrdal & Kim, 2008). The fact that we find a tighter coupling of F0 values between speakers than would be expected by chance suggests that, although questions and responses would be considered separate speech acts (Holtgraves, 2008; Levinson, 2017; Searle, 1969), the sequential organization of these speech acts, in which questions directly elicit a pragmatically fitting response, provides a layer of contingency and, thus, a “binding relationship” between the question and the response.

### Interpersonal entrainment as a two-person problem

The main focus of our study was on the association between social predispositions and entrainment. We found associations between kinematic entrainment of both the head and hands with partner differences in EQ score. We initially hypothesized that such an association would be negative, such that higher EQ-score differences would be associated with lower behavioral entrainment. Our findings of higher entrainment associating with higher EQ-score difference is thus in contrast with the pattern expected by the double-empathy problem—that motor similarity would be lower in dyads with greater differences in empathy. Thus, the present study did not support the theory that the patterns expected by the double-empathy problem would extend to motor entrainment, at least in the sense of movement similarity during questions and their responses. When considering why the present study and that by Georgescu et al. (2020) found no evidence for the patterns expected by the double-empathy problem for motor entrainment, it should be noted that the hypotheses of both studies took high entrainment (i.e., low behavioral dissimilarity) to be a positive social outcome, reached with successful social interaction.

Entrainment may be more appropriately viewed as a tool to promote better social outcomes, as entraining behaviors between individuals have been found to be beneficial for various social outcomes (Bowsher-Murray et al., 2023; Galbusera et al., 2019; Hove & Risen, 2009; Rabinowitch & Meltzoff, 2017; Tarr et al., 2015; Vacharkulksemsuk & Fredrickson, 2012). Therefore, entrainment may increase with increasing EQ score differences between conversational partners in order to promote better social outcomes, as a compensatory mechanism for interpersonal differences. When considered within the logic of the double-empathy problem and Williams et al.’s (2021) finding that communication is more successful between people who are more similar to each other, it is possible that communication between those participants who differed more from each other was more difficult initially. Thus, to promote better social outcomes, participants who differed more from their partners may have spontaneously increased entrainment to promote more-positive social outcomes, compensating for their interpersonal differences on the EQ.

This putative compensatory mechanism follows past findings that head-movement entrainment increases with increasing conversation difficulty (Hadley & Ward, 2021). Such a compensatory mechanism leads to the question of why entrainment would not remain at its greatest in all conversations to achieve the best possible social outcomes. While the majority of interpersonal entrainment research has investigated the benefits of entrainment, few studies have investigated the disadvantages of increased entrainment (Galbusera et al., 2019). Galbusera et al. (2019) found that, while motor entrainment increased social affect, it also reduced self-regulation of affect—that is, higher motor entrainment was associated with lower self-reported ability to (prereflectively) regulate or modulate one’s own emotions in the moment. Thus, entrainment may be used to compensate for increased conversational difficulty when interpersonal differences are high but reduced when such differences are less prevalent in order to protect intrapersonal processes like self-regulation. Similarly, Wallot et al. (2016) found that lower interpersonal entrainment was associated with better performance on a complex joint-action task (Wallot et al., 2016), again highlighting the importance of behavioral dissimilarity in certain contexts. This mechanism also fits well with the framing of dialogue as interpersonal synergy (Fusaroli et al., 2014), wherein both entrainment and divergence are important aspects of interaction. Of course, future research should also investigate whether the relationship between kinematic entrainment and EQ differences holds in other conversational turn sequences or whether it is specific to question–response sequences.

We found that, contrary to our hypothesis, no significant relationship between EQ score differences and prosodic dissimilarity. Interestingly, the prosodic results did not follow the same pattern as the motor results. One potential explanation is that acoustic entrainment, when visual entrainment is present, may be redundant. Human communication is multimodal, and interference with either the visual or the audio signal can be compensated for by the presence of the other (Alexanderson & Beskow, 2014; Davis et al., 2006; Drijvers & Özyürek, 2017; Drijvers et al., 2018). As motor entrainment was already increasing with EQ score differences, such an increase in prosodic matching may have been redundant, as the full visual signal was available. This theory follows synergy theories of how multimodal forms of entrainment come together. In these framings, entrainment often does not extend to all modalities (Dale et al., 2013; Esteve-Gibert et al., 2022; Fusaroli et al., 2014; Fusaroli & Tylén, 2016; Trujillo et al., 2023). This is also in line with research showing that compensatory exaggeration of one modality, such as speaking louder in noisy environments, does not always go hand-in-hand with exaggeration of other modalities (Garnier et al., 2018; Trujillo et al., 2022, 2021). Given that the current study focused on question–answer sequences, it is also possible that prosodic entrainment and EQ differences are present in other aspects of conversation, or even correspond to a different time scale (e.g., occurring as a general trend over the course of conversation but not specifically according to turn sequences).

Finally, our exploratory post hoc analysis showed no evidence for a strong association, whether positive or negative, between kinematic entrainment and prosodic entrainment. This is contrary to what was found between kinematic and some levels of linguistic entrainment in a previous study on Danish and Norwegian conversation (Trujillo et al., 2023). This is particularly interesting given that F0 entrainment in real pairs was stronger than in pseudo-pairs. This means that while kinematic entrainment is more strongly associated with interpersonal differences in self-reported EQ, it may be relatively independent of the entrainment that we observe in speech prosody. Future analyses will be needed to determine the extent to which and the time scales according to which, prosodic entrainment relates to interpersonal trait differences.

### Future directions and practical applications

Future research should assess whether other personality traits, or social predispositions, are also predictive of interpersonal coordination. One possibility is that empathy quotient scores form just one part of a cluster of personality traits that are associated with interpersonal coordination. For example, empathy, extraversion, and agreeableness are positively associated with facial mimicry in simple facial expression viewing paradigms (Perugia et al., 2020; Rymarczyk et al., 2019). An interesting direction for future work would be to quantify the (potential) network of interpersonal trait differences that contribute to the extent of coordination. This avenue of research could be useful both for developing a stronger theoretical understanding of interpersonal coordination, and to inform data-driven–team-management decisions (i.e., predicting who will work best with whom).

It should be noted that the current analyses focused on conversations between speakers who were already acquainted with one another. An interesting future direction would be to test whether the current results generalize to other types of relationships. Additionally, future research should assess whether the associations between EQ and entrainment are apparent very early in a conversation between people who do not yet know each other or if the association is the result of a mutual adaptation that occurred over a longer time period.

While the current study has identified EQ differences as a factor related to the extent of entrainment between two individuals, future studies should additionally investigate whether differences in other traits also impact multimodal entrainment. As the EQ does not distinguish between different types of empathy, future research should utilize a more-refined measure with subscales for cognitive and affective empathy. This is particularly important as the two studies that had found a relationship between interpersonal entrainment and empathy had used different measures of empathy (Fujiwara & Daibo, 2022; Zampella et al., 2020). Future research, investigating the development of familiarity and (potential) parallel development of motor entrainment, could elucidate how differences in empathy scores might predict the longer-scale development of stable interpersonal motor entrainment patterns among interlocutors who are not already familiar with one another.

While this research was inspired by autistic-person’s differences in interpersonal synchrony, our transdiagnostic approach meant we did not investigate this population, specifically. It is hoped that in contributing to an understanding of the mechanisms of interpersonal synchrony, evidence-based advice can be produced to support populations who demonstrate differences in interpersonal synchrony, such as, but not necessarily limited to, autistic people. Such investigations into entrainment mechanisms are prudent because entrainment is associated with various positive social outcomes both in autistic people (Manders et al., 2022) and the wider population (Bowsher-Murray et al., 2023; Hove & Risen, 2009; Rabinowitch & Meltzoff, 2017; Tarr et al., 2015; Vacharkulksemsuk & Fredrickson, 2012). Positive social outcomes are important for various aspects of daily life—including job interviews (Strickland et al., 2013), which have been implicated as one of the potential barriers (Kumazaki et al., 2019) resulting in the underhiring and underrepresentation of autistic people in the workforce (Taylor & Seltzer, 2011). Understanding the mechanisms of reduced synchrony in this population could facilitate the development of inclusive policies for hiring and for other areas of life wherein autistic people are being systematically disadvantaged by current policies; for example, employers could receive education about reduced entrainment in autistic candidates and be encouraged to consciously consider whether this is influencing their hiring decisions.

In line with our proposal that interpersonal synchrony may work to facilitate positive social interactions, methods such as dance movement therapy, in which dance partners mirror each other, have been found to longitudinally improve social affect and affective engagement (Manders et al., 2022). Thus, group environments in which autistic people experience social exclusion, such as classrooms (Rotheram-Fuller et al., 2010), could utilize such methods to facilitate positive social affect among members. While these applications have been discussed specifically in relation to autistic individuals, differences in entrainment and reduced social outcomes might also be present in other (diagnosed or undiagnosed) neurodivergent groups (Quintero et al., 2019; Wehmeier et al., 2010). By better understanding what underlying interpersonal differences lead to such group differences, these applications can be more appropriately employed.

Finally, it is important to note that, because we focused on question–response sequences, any entrainment occurring between speakers is likely primarily driven by the responder matching the behavior of the questioner. In other words, within the level of dialogue acts (i.e., questions and responses, in this case), entrainment will be largely unidirectional, with the former influencing the latter. However, this does not mean that the entrainment value of a given dyad is based on one particular speaker entraining to the other. As both speakers asked questions and gave responses, the entrainment values essentially reflect entrainment in both directions, in terms of Speaker A entraining to Speaker B and vice versa.

## Conclusions

Our study found that greater EQ differences between interlocutors are associated with greater kinematic entrainment during question–response sequences. Entrainment may, therefore, serve to compensate for increased interpersonal differences in empathy. Our findings provide new insights into how interpersonal coordination is associated with interpersonal differences in social traits.
